# Meta-Analysis of the Use of 8-OHdG in Saliva as a Marker of Periodontal Disease

**DOI:** 10.1155/2018/7916578

**Published:** 2018-05-02

**Authors:** Esther Paredes-Sánchez, José María Montiel-Company, José Enrique Iranzo-Cortés, Teresa Almerich-Torres, Carlos Bellot-Arcís, José Manuel Almerich-Silla

**Affiliations:** Departamento de Estomatología, Facultad de Medicina y Odontología, Universidad de Valencia, C/ Gascó Oliag núm. 1, 46010 Valencia, Spain

## Abstract

The objective was to collect the available evidence on oxidative stress marker measurements in periodontal patients, focusing specifically on 8-hydroxy-2′-deoxyguanosine (8-OHdG) as a salivary marker of periodontal disease, and to perform meta-analyses to calculate differences in concentration compared to healthy persons. A systematic search in PubMed, Cochrane Library, Embase, and Scopus identified 81 articles. Of these, 38 were duplicates. After reading the abstracts of the remaining 43, 42 were selected for full-text assessment. Finally, 17 articles were included in the qualitative synthesis. Those excluded were of low quality, did not answer the research question, or did not meet the inclusion and exclusion criteria. Of the 17 in the qualitative synthesis, 9 were included in the meta-analysis. The 9 studies in the meta-analysis were combined in a random effects model. Their heterogeneity was high (*Q* = 3982.02, *p* < 0.001, *I*^2^ = 99.8%). The difference in mean 8-OHdG concentration in saliva between periodontal and healthy subjects was estimated at 2.11 ng/ml (95% CI 1.23–2.98). The different saliva collection methods (stimulated/unstimulated) did not explain the heterogeneity. The 8-OHdG levels in saliva of periodontal patients were almost double to those of healthy patients: 8-OHdG is clearly a powerful periodontal disease marker.

## 1. Introduction

Periodontal disease (PD) is a chronic inflammatory disorder that affects 10–15% of the world population and is considered the greatest cause of tooth loss, causing damage to all the structures that support the teeth: periodontal ligament, root cement, alveolar bone, and gingival tissues [1–4]. Its clinical classification is based on the presence or absence of signs of inflammation, periodontal pocket depth, gingival attachment loss, and bone loss [1, 2].

As it progresses, neutrophils at the site increase and, associated with macrophages, produce cytosines such as tumour necrosis factor alpha (TNF-*α*), interleukin-1 (IL-1), and prostaglandins [1]. During this inflammatory process, fibroblasts are stimulated by interleukin-1 and extracellular matrix metalloproteinases (MMPs) are secreted, particularly collagenase produced by polymorphonuclear neutrophils [1].

MMPs cause collagen degradation and TNF-*α* is responsible for increased osteoclast activity, leading to bone resorption. In addition, T-lymphocytes secrete the receptor activator of nuclear factor *κ*B ligand (RANKL), which, in turn, is involved in osteoclast activity, ending in bone loss [1]. Polymorphonuclear lymphocytes (PMNLs) are believed to produce active reactive oxygen species (ROS) and therefore to lead to greater production of these species [4].

Periodontal disease progression depends on immune response and the host's susceptibility [2]. Numerous studies have pointed out that both oxidative stress and the total antioxidant capacity of the individual play an important role in the pathogenesis of periodontal diseases [2, 3]. It has been shown that reduced antioxidant concentrations in the gingival crevicular fluid (GCF) help to increase the damage to the gums and surrounding structures caused by the action of the neutrophils [3]. In the same way, several recent studies have shown that chronic periodontal disease is associated with hyperreactive neutrophils that have increased the production of reactive oxygen species as a response to stimulation of the Fc-gamma receptor [4].

Oxidative stress is defined as the state in which the balance between prooxidants and antioxidants in the organism is disturbed [1, 2, 5]. This imbalance is caused by an excess of reactive oxygen species, free radicals, and other reactive molecular species and/or by a deficiency in the antioxidant mechanisms arising from direct or indirect damage to the tissues [2, 3, 5].

One of the most important markers of oxidative stress is 8-hydroxy-2′-deoxyguanosine (8-OHdG), which is formed through oxidation of guanine from damaged DNA [2, 5]. Numerous studies have observed higher 8-OHdG levels in the saliva of subjects with periodontal disease than in that of healthy subjects, showing that this marker is correlated with increased ROS production during periodontal inflammation [2, 3, 5]. In the same way, its levels fall when periodontitis patients receive successful anti-inflammatory treatment [5]. Almerich-Silla et al. [6] showed a high correlation between the presence of periodontal bacteria and the levels of 8-OHdg in saliva, which they found to be far higher than those of other oxidative stress markers.

During chronic inflammation, not only do reactive oxygen species increase in the affected tissues but a reduction in antioxidant levels is also observed [5]. Antioxidants can be defined as substances which, at low concentrations with respect to the oxidisable substrate, significantly reduce or inhibit oxidation of that substrate [4]. They combat oxidative damage through direct elimination of ROS and repair of the damage caused by these detrimental agents. Antioxidants also act by downregulating some redox-sensitive proinflammatory gene transcription factors and, simultaneously, regulating inflammatory gene transcription factors [2].

Antioxidants are classified according to their mode of action. The preventive antioxidants include superoxide dismutase (SOD) enzymes, catalase (CAT), glutathione peroxidase (GPx), glutathione reductase (GR), and DNA repair enzymes. The eliminating antioxidants include ascorbate (vitamin C), carotenoids (including retinol—vitamin A), uric acid, *α*-tocopherol (vitamin E), and polyphenols (flavonoids) [4].

Saliva has an important role as a tool for diagnosing and predicting periodontal diseases [3]. Saliva is defined as an accessible bioxide which contains components derived from the oral mucus surfaces, gingival crevices, and tooth surfaces. It contains microorganisms that colonise the mouth, and other exogenous substances, and can therefore provide a picture of the host's relation to the environment.

Over 2000 proteins and enzymes have been identified in saliva. They include aspartate amino transferase (AST), the level of which is positively correlated to the intensity and extent of periodontal inflammation. The same is true of proteinases, lactoferrin, and metalloproteinases.

One of the proteins present in saliva is C-reactive protein (CRP), a known indicator of inflammatory activity. Its levels increase during periodontal disease and fall when anti-inflammatory treatment is successful [5].

Biomarker determination in saliva is becoming an important part of laboratory diagnosis and the prediction of periodontal diseases, and more studies are needed to acquire greater knowledge in this field [4].

The objective of this study was to collect all the available evidence in scientific publications on measurements of oxidative stress markers in persons with periodontal disease, concentrating specifically on 8-OHdG as a salivary marker of disease, and conduct a meta-analysis to calculate the difference in salivary concentration of this marker between healthy persons and patients with periodontal disease.

## 2. Materials and Methods

To accomplish the objective of this systematic review, a focused research question was formulated with the following components:

- Case: patients with periodontal disease
- Comparison: between patients with periodontal disease and healthy subjects
- Result: concentration of 8-OHdG in saliva.

### 2.1. Search Strategy and Article Selection

To identify the most relevant studies irrespective of language, searches were made in the PubMed, Cochrane, Scopus, and Embase databases in March 2017. The search strategy was based on combination of the following key words: “Periodontal disease” AND “Oxidative stress markers” AND “Saliva”.

Two calibrated reviewers (EP-S and JMM-C) independently selected the articles. In the event of disagreement, they had to reach a consensus on which articles to include or to exclude from the review. Cohen's kappa was used to measure interexaminer reliability (kappa = 0.85). The initial screening was performed by reading the titles and abstracts. If the information was insufficient, the decision was taken after reading the full text.

### 2.2. Inclusion and Exclusion Criteria

The inclusion criteria were all articles identified in the database searches, filtered by “humans” but without filtering by publication date or age of subjects, and no article was rejected for language reasons.

Literature reviews were excluded, as were studies linking periodontal disease to other types of systemic disease, studies of pregnant women, and studies of children or adolescents. Studies that did not address periodontal disease, lacked a control group, or examined variables other than the objective of the present review were also excluded.

### 2.3. Variables Recorded

The following data were collected for each article: author and year of publication, type of study, sample size, gender and age, the variables studied (different reactive oxygen species and antioxidants such as 8-OHdG, AST, ALP, ALT, uric acid, cortisol, TAC, MDA, Gpx, SOD, GR and CAT), diagnostic criteria for periodontal disease, saliva collection method, 8-OHdG levels in the control and periodontal disease groups, and study quality.

### 2.4. Quality Assessment

The quality of each case-control study was measured on the Newcastle-Ottawa quality assessment scale for case control studies (NOS). This consists of 8 items, divided into three groups: selection of study groups, comparability between cases and controls, and exposure or interesting finding in the case group and control group, respectively. The stars awarded for each quality group provide a rapid visual assessment. The scoring system can award a maximum of 10 stars to studies of the highest quality.

### 2.5. Statistical Analysis

The measure of effect for the meta-analyses was the difference in mean 8-OHdG concentration between the periodontal patients and the control group. The studies were combined using the random effects model. The significance of the effect estimate was measured with the *Z* test when *p* value < 0.05. Heterogeneity was measured by the *p* value of the *Q* test and by *I*^2^. A *Q* test *p* value of under 0.1 was considered to show heterogeneity, which was classed as mild when the *I*^2^ result was between 25% and 50%, moderate when within 50–75%, and high when over 75%. The meta-analyses were represented graphically by forest plots. The publication bias was represented graphically by a funnel plot and the classic fail-safe number and Egger's regression intercept and its *p* value were calculated.

## 3. Results

### 3.1. Flow Chart

**Figure 1:**
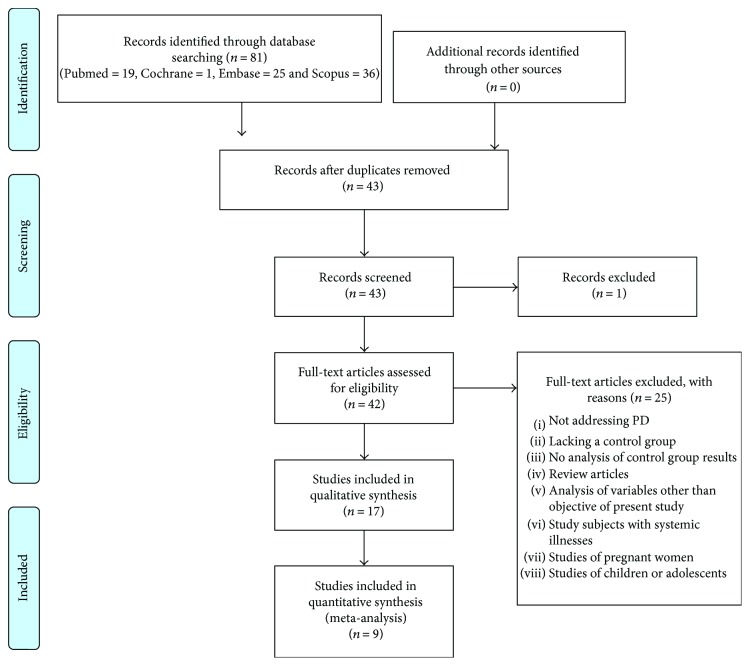
Flow chart.

Searching in the databases resulted in 19 articles identified in PubMed, 1 in the Cochrane Library, 25 in Embase, and 36 in Scopus, totalling 81 articles. Of these, 38 were duplicates and were removed. After reading the titles and abstracts of the remaining 43, 42 were selected for full-text assessment, which reduced their number to 17 articles (Figure 1). The reasons for exclusion were: not addressing periodontal disease (PD), lack of a control group, lack of analysis of the control group results, review articles, analysing variables other than those of the present study objective, study subjects with systemic illnesses, studies of pregnant women, and studies of children or adolescents.

A total of 17 articles were included in the qualitative synthesis. All were case-control studies. Subsequently, 9 articles were selected for quantitative synthesis.

### 3.2. Study Quality

The study quality findings are shown in Table 1. Of the 17 studies included in the qualitative synthesis, 2 studies scored 9 stars out of 10 on the NOS, 10 studies scored 8 out of 10, 2 studies scored 7 out of 10, and finally, 2 studies scored 5 out of 10 (Table 1).

### 3.3. Qualitative Analysis

The qualitative synthesis included 17 studies (Table 2). Their sample size ranged from 35 to 160 participants, aged between 16 and 82 years. The criteria employed to diagnose both the presence of periodontal disease and the absence of both gingivitis and periodontitis were as follows: periodontitis when a minimum of 2 teeth had pocket depths of 4 millimetres or more, absence of gingivitis and periodontitis when the participant presented no history of periodontal disease, no gingival inflammation, and good oral hygiene. Both stimulated and unstimulated saliva samples were included in the analysis. They were centrifuged, frozen, and stored at minus 80°C. Out of the 17 studies, 10 used 8-OHdG as an oxidative stress marker.

### 3.4. Quantitative Analysis

Of the 10 studies that used 8-OHdG as an oxidative stress marker, one was removed from the meta-analysis due to a very low Newcastle-Ottawa Scale (NOS) score (5 stars out of 10). The remaining 9 studies attained a minimum quality score of 7 stars on the NOS and compared periodontal patients with healthy controls or with patients with gingivitis. To examine the mean 8-OHdG levels in healthy subjects and how they differed from those of patients with periodontal disease, several meta-analyses were performed, combining the studies by means of the random effects model.

**Figure 2:**
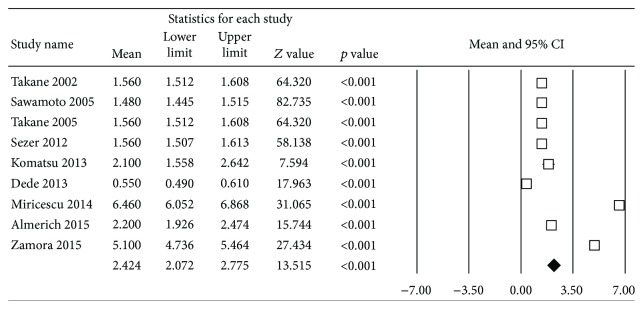
Forest plot of mean 8-OHdG concentration in healthy subjects.

To estimate the 8-OHdG concentration in healthy subjects (Figure 2), 9 studies were included in the meta-analysis. When combined, they showed high heterogeneity (*Q* test = 1924, *p* ≤ 0.001, *I*^2^ = 99.6%). The levels of 8-OHdG in saliva of the healthy individuals were estimated as 2.42 ng/ml with a 95% confidence interval of 2.07–2.78 ng/ml.

**Figure 3:**
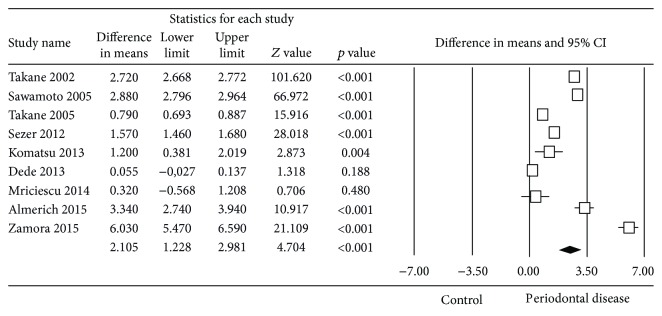
Forest plot of the differences in mean 8-OHdG concentration between periodontal disease patients and healthy controls.

The estimated difference in mean salivary concentration of 8-OHdG between healthy subjects and patients with periodontal disease (Figure 3) was 2.11 ng/ml, with a 95% confidence interval of 1.23–2.98, showing that the concentration was significantly higher in the periodontal disease patients (*Z* test = 4.70, *p* ≤ 0.001). The meta-analysis presented high heterogeneity (*Q* test = 4188.3, *p* ≤ 0.001, *I*^2^ = 99.81%).

**Figure 4:**
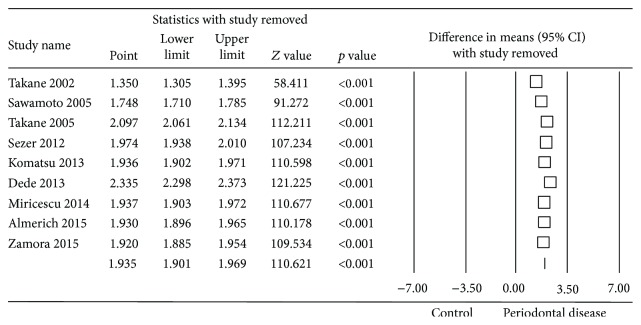
Forest plot of the differences in mean 8-OHdG concentration between periodontal disease patients and healthy controls using the “one study removed” method.

To assess the sensitivity of the meta-analysis, the “one study removed” method was employed. This found that removing each study in turn from the meta-analysis barely altered the difference in means obtained. Removal of Dede et al. study [7] could overestimate the estimation obtained, although not significantly (Figure 4).

**Figure 5:**
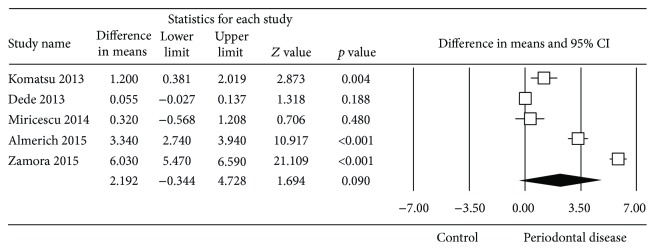
Forest plot of the differences in mean 8-OHdG concentration between periodontal disease patients and healthy controls with unstimulated saliva sample collection.

**Figure 6:**
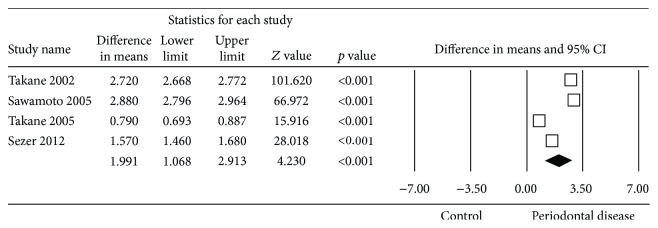
Forest plot of the differences in mean 8-OHdG concentration between periodontal disease patients and healthy controls with stimulated saliva sample collection.

To examine the possible sources of heterogeneity, the effect of the saliva sample collection method was assessed by analysing the studies which used stimulated saliva separately from those that did not. These two meta-analyses showed that the heterogeneity persisted in both groups (Figures 5 and 6).

### 3.5. Publication Bias

**Figure 7:**
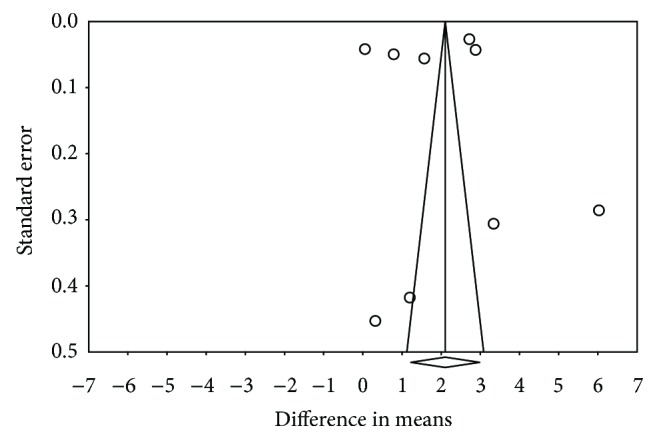
Funnel plot.

On examining the funnel plot of the studies included in the meta-analysis, a certain asymmetry was observed (Figure 7). However, Egger's regression intercept gave a value of −2.76 with a *p* value of 0.84, indicating the absence of publication bias. In addition, the classic fail-safe number—the number of studies that would be needed for a significant meta-analysis to lose its significance—was 6190 studies, which indicates a very low risk of publication bias.

## 4. Discussion

Periodontitis is an irreversible inflammatory disease that affects the tissues supporting the teeth. Once initiated, it progresses with the loss of collagen fibres and of attachment to the surface of the cement, apical migration of the pocket epithelium, and resorption of the alveolar bone. If not treated at an early stage, the disease advances to progressive destruction of the bone, leading to movement of the teeth and their subsequent loss [8].

Inflammatory and immune reactions to the bacterial plaque perform the leading roles in the pathogenesis of periodontitis. Most of the tissue destruction is considered to be the result of impairment of the inflammatory and immune response to this microbial plaque, causing the liberation of neutrophils, reactive oxygen species, and enzymes [8].

A large number of distinct types of bacteria with different pathogenicity increase periodontal inflammation. ROS are related to PMN action in the destruction of periodontal pathogens. This rise of ROS levels by PMNs would lead to tissue degeneration and a worse status of periodontal disease. Salivary stress parameters of 8-OHdG are correlated with periodontal disease and *Porphyromonas gingivalis* and its genotypes fimA II and Ib, *Treponema denticola*, *Aggregatibacter actinomycetemcomitans*, and *Tannerella forsythia* [6].

Numerous studies have pointed out that both oxidative stress and the individual's total antioxidant capacity are disturbed in subjects with periodontal disease, showing the existence of a direct association between the rise in reactive oxygen species and the fall in total antioxidant capacity in the pathogenesis of periodontal disease [2, 3, 8, 9]. The imbalance between oxidants and antioxidants have been related with the destruction of the periodontium during inflammatory periodontal [10, 11].

The use of some antioxidants (lycopene and vitamin E) as periodontal treatment has the potential to improve periodontal clinical parameters; nevertheless, the role of antioxidant/oxidative stress parameters needs further investigations [1].

Miricescu et al. [8] studied the relations between the antioxidant defence system of saliva and the levels of reactive oxygen species (ROS) in patients with chronic periodontitis and in subjects free of periodontal disease. They observed significantly higher ROS values in the chronic periodontitis group than in the control group and significantly lower levels of certain antioxidants such as uric acid, TAC, and Gpx.

8-Hydroxy-2′-deoxyguanosine (8-OHdG) is formed through oxidation of guanine from damaged DNA, causing severe damage to periodontal tissues [2, 5]. Higher salivary 8-OHdG reflect increased oxygen radical activity during periodontal inflammation [6]. The present review has shown that although 8-OHdG is present both in subjects with no periodontal disease and in those with this illness, its levels are significantly higher in the saliva of the periodontal disease patients [6, 12–14].

Takane et al., Sawamoto et al., Sezer et al., Komatsu et al., Almerich-Silla et al., and Zamora-Perez et al. all found the 8-OHdG levels in subjects with periodontal disease to be high, and very high compared to those in healthy controls. The findings of all these studies were statistically significant [6, 12, 14–17]. Of all the studies examined, only Takane et al. and Dede et al. did not present statistically significant results, possibly owing to bias in saliva sample collection and also to using different periodontal disease classification criteria [7, 13].

In the present review, the quantitative synthesis of 9 studies found that the reactive oxygen species 8-OHdG is an oxidative stress marker that presents higher levels in the saliva of subjects with periodontal disease than in healthy controls. The raised concentration of this reactive oxygen species in the population with periodontal disease was estimated as 2.11 ng/ml higher compared to that in the controls, with a confidence interval of 1.12–2.98 ng/ml. However, high heterogeneity was observed between the studies included in the meta-analysis, so these findings should be treated with caution.

To investigate the possible causes of the observed heterogeneity, separate meta-analyses were performed by saliva collection method (stimulated or unstimulated). It was found that the heterogeneity persisted irrespective of the collection method, though all the studies showed a positive association between 8-OHdG levels in saliva and periodontal disease. Having rejected the saliva collection method as the cause, other possible sources of heterogeneity could be the method used to analyse the saliva, the criteria for classifying periodontal disease, or even differences between the populations studied.

Different methods were employed to assess the possible publication bias. The classic fail-safe number, which estimates the number of studies that would be required for the meta-analysis to cease to be significant, returned a value of 6190, which is very high. This shows that despite the asymmetry of the funnel plot, the present meta-analysis could have little exposure to publication bias, a finding that was reinforced by the Egger's regression intercept *p* value of 0.84.

When selecting the studies, the Newcastle-Ottawa quality assessment scale for case control studies (NOS) was used to determine their quality. This scale classes the studies as being of high, medium, or low quality according to the number of items for which they are awarded points for meeting the selection, comparability, and exposure criteria. Of the 10 studies that used 8-OHdG, only one was removed from the meta-analysis due to a very low NOS score (5 stars out of 10).

## 5. Conclusions

The present meta-analysis estimated that the concentration of 8-OHdG in saliva of the subjects with periodontal disease was 2.11 ng/ml higher than that of healthy subjects, almost double the concentration in the latter, with a 95% confidence interval of 1.12–2.98. As a result, it may be stated that there is clear evidence that 8-OHdG is a powerful marker of periodontal disease.

## Figures and Tables

**Table 1:** Quality of the studies according to the Newcastle-Ottawa quality assessment scale.

Study	Selection	Comparability	Exposure	Total stars
Takane et al. 2002 [12]	^∗∗∗∗^	^∗^	^∗∗∗^	8
Sawamoto et al. 2005 [15]	^∗∗∗∗^	^∗^	^∗∗∗^	8
Takane et al. 2005 [13]	^∗∗∗∗^	^∗^	^∗∗∗^	8
Totan et al. 2006 [18]	^∗∗∗∗^	^∗^	^∗∗∗^	8
Greabu et al. 2006 [9]	^∗∗∗^	^∗^	^∗∗^	6
Badea et al. 2010 [19]	^∗∗∗∗^	^∗^	^∗∗^	5
Sezer et al. 2012 [14]	^∗∗∗∗^	^∗∗^	^∗∗∗^	9
Komatsu et al. 2013 [16]	^∗∗∗^	^∗^	^∗∗∗^	7
Dede et al. 2013 [7]	^∗∗∗∗^	^∗^	^∗∗∗^	8
Pendyala et al. 2013 [20]	^∗∗∗∗^	^∗^	^∗∗∗^	8
Miricescu et al. 2014 [8]	^∗∗∗∗^	^∗^	^∗∗∗^	8
Hernández-Monjaraz et al. 2014 [21]	^∗∗^	^∗^	^∗∗^	5
Baňasová et al. 2014 [22]	^∗∗∗∗^	^∗^	^∗∗∗^	8
Baltacioglu et al. 2014 [23]	^∗∗∗∗^	^∗^	^∗∗∗^	8
Trivedi et al. 2014 [24]	^∗∗∗∗^	^∗^	^∗∗∗^	8
Almerich-Silla et al. 2015 [6]	^∗∗∗∗^	^∗∗^	^∗∗∗^	9
Zamora-Perez et al. 2015 [17]	^∗∗∗∗^	^∗∗^	^∗∗∗^	9

**Table 2:** Qualitative analysis.

Study author/year [reference]	Study type	*n*; age (years)	Variables	Case criteria (PD)	Saliva sample	Results 8-OHdG (ng/ml)	Significant difference	NOS
Takane et al. 2002 [12]	CC	PD: 78; 21–69 C: 17; 24–64	8-OHdG	At least 2 sites with PP > 4 mm	S	PD 4.28 ± 0.10 C 1.56 ± 0.10	Yes	8/10
Sawamoto et al. 2005 [15]	CC	PD:29; 36–68 C: 20; 25–65	8-OHdG	At least 2 sites with PP > 4 mm	S	PD 4.36 ± 0.18 C: 1.48 ± 0.08	Yes	8/10
Takane et al. 2005 [13]	CC	PD: 18; 21–67 C: 17; 24–64	8-OHdG	At least 2 sites with PP > 4 mm	S	PD 2.35 ± 0.18 C 1.56 ± 0.1	Yes	8/10
Totan et al. 2006 [18]	CC	PD: 25; 25–55 C: 25; 25–55	AST ALT ALP	PP > 5 mm, LA > 40%	NS	—	—	8/10
Greabu et al. 2006 [9]	CC	PD:30; 16–82 C: 6; 16–82	Uric acid Cortisol TAC	Not specified	NS	—	—	6/10
Badea et al. 2010 [19]	CC	PD: 85; — C: 49; —	8-OHdG	Not specified	—	PD 3.00–7.50 C 1.20–1.85	Yes	5/10
Sezer et al. 2012 [14]	CC	PD: 20; 29–58 G: 20; 29–58 C: 20; 29–55	8-OHdG	At least 4 sites with PP > 5 mm	S	PD 3.13 ± 0.22 G 1.58 ± 0.13 C 1.56 ± 0.12	Yes (Not for PD versus G)	9/10
Komatsu et al. 2013 [16]	CC	PD: 21; 44.7 ± 8.8 C: 26;41.9 ± 6.2	8-OHdG	PDT sensor probe	NS	PD 3.30 ± 1.44 C 2.10 ± 1.41	Yes	7/10
Dede et al. 2013 [7]	CC	PD: 24; 30–55 C: 24; 18–30	8-OHdG	At least 2 sites with PP > 5 mm and LB > 30%	NS	PD 0.61 ± 0.14 C 0.50 ± 0.15	No	8/10
Pendyala et al. 2013 [20]	CC	PD: 30; 40–65 C: 30; 40–65	TAC	2 or more sites with PP > 4 mm or LA > 4 mm with bleeding	NS	—	—	8/10
Miricescu et al. 2014 [8]	CC	PD: 20 C: 20 51.3 ± 7.4	8-OHdG MDA TAC GPx	At least 6 sites with PP > 4 mm, LB > 30%	NS	PD 6.78 ± 1.80 C 6.46 ± 0.93	No	8/10
Hernández-Monjaraz et al. 2014 [21]	CSt	PD: 23 C: 26	SOD TBARS TAC	Not specified	—	—	—	5/10
Baňasová et al. 2014 [22]	CC	PD: 23; 43 ± 7.6 C: 19; 39.1 ± 8.8	TBARS	At least 16 sites with PP > 4 mm in 2 different quadrants	S	—	—	8/10
Baltacioglu et al. 2014 [23]	CC	PD: 68; 26–42 C: 30; 26–37	MDA TOS OSI TAOC	Multiple sites with PP > 5 mm, LB > 30% and LA > 5 mm.	NS	—	—	8/10
Trivedi et al. 2014 [24]	CC	PD: 30; 20–65 C: 30; 20–65	SOD GR CAT MDA	At least 2 sites with PP > 4 mm or LA > 4 mm	NS	—	—	8/10
Almerich-Silla et al. 2015 [6]	CC	PD: 33; 41–45 G:16; 35–43 C:37; 38–43	8-OHdG MDA GPx SOD TAOC	At least 4 sites with PP > 5 mm and LA > 2 mm	NS	PD 5.54 (4.96–6.12); G 2.33 (1.84–2.82); C 2.20 (1.88–2.52)	Yes (Not for G versus C)	9/10
Zamora-Perez et al. 2015 [17]	CC	PD: 100; 39.03 C: 60; 40.13	8-OHdG	At least 6 sites with PP >6 mm and LA >5 mm	NS	PD 11.13 ± 1.91 C 5.10 ± 1.44	—	9/10

*N*: sample size; CC: case-control; CS: cross-sectional; PD: periodontal disease; G: gingivitis; C: control group; PP: periodontal pocket; LA: loss of attachment; LB: level of bleeding; S: stimulated; NS: not stimulated.

## References

[1] Muniz F. W. M. G., Nogueira S. B., Mendes F. L. V. (2015). The impact of antioxidant agents complimentary to periodontal therapy on oxidative stress and periodontal outcomes: a systematic review.

[2] Buczko P., Zalewska A., Szarmach I. (2015). Saliva and oxidative stress in oral cavity and in some systemic disorders.

[3] Trivedi S., Lal N. (2017). Antioxidant enzymes in periodontitis.

[4] Podzimek S., Vondrackova L., Duskova J., Janatova T., Broukal Z. (2016). Salivary markers for periodontal and general diseases.

[5] Henry L. G., McKenzie R. M., Robles A., Fletcher H. M. (2012). Oxidative stress resistance in *Porphyromonas gingivalis*.

[6] Almerich-Silla J. M., Montiel-Company J. M., Pastor S., Serrano F., Puig-Silla M., Dasi F. (2015). Oxidative stress parameters in saliva and its association with periodontal disease and types of bacteria.

[7] Dede F. Ö., Özden F. O., Avcı B. (2013). 8-hydroxy-deoxyguanosine levels in gingival crevicular fluid and saliva in patients with chronic periodontitis after initial periodontal treatment.

[8] Miricescu D., Totan A., Calenic B. (2014). Salivary biomarkers: relationship between oxidative stress and alveolar bone loss in chronic periodontitis.

[9] Greabu M., Purice M., Totan A., Spînu T., Totan C. (2006). Salivary cortisol-marker of stress response to different dental treatment.

[10] Waddington R. J., Moseley R., Embery G. (2000). Reactive oxygen species: a potential role in the pathogenesis of periodontal diseases.

[11] Borges I., Moreira E. A. M., Filho D. W., de Oliveira T. B., da Silva M. B. S., Fröde T. S. (2007). Proinflammatory and oxidative stress markers in patients with periodontal disease.

[12] Takane M., Sugano N., Iwasaki H., Iwano Y., Shimizu N., Ito K. (2002). New biomarker evidence of oxidative DNA damage in whole saliva from clinically healthy and periodontally diseased individuals.

[13] Takane M., Sugano N., Ezawa T., Uchiyama T., Ito K. (2005). A marker of oxidative stress in saliva: association with periodontally-involved teeth of a hopeless prognosis.

[14] Sezer U., Çiçek Y., Çanakçi C. F. (2012). Increased salivary levels of 8-hydroxydeoxyguanosine may be a marker for disease activity for periodontitis.

[15] Sawamoto Y., Sugano N., Tanaka H., Ito K. (2005). Detection of periodontopathic bacteria and an oxidative stress marker in saliva from periodontitis patients.

[16] Komatsu T., Duckyoung Y., Ito A. (2013). Increased oxidative stress biomarkers in the saliva of Down syndrome patients.

[17] Zamora-Perez A. L., Ortiz-Garcia Y. M., Lazalde-Ramos B. P. (2015). Increased micronuclei and nuclear abnormalities in buccal mucosa and oxidative damage in saliva from patients with chronic and aggressive periodontal diseases.

[18] Totan A., Greabu M., Totan C., Spinu T. (2006). Salivary aspartate aminotransferase, alanine aminotransferase and alkaline phosphatase: possible markers in periodontal diseases?.

[19] Badea V., Balaban D., Amariei C., Nuca C., Bucur L. (2010). Salivary 8-hidroxy-2-deoxy guanosine as oxidative stress biomarker for the diagnosis of periodontal disease.

[20] Pendyala G., Thomas B., Joshi S. R. (2013). Evaluation of total antioxidant capacity of saliva in type 2 diabetic patients with and without periodontal disease: a case-control study.

[21] Hernández-Monjaraz B., Ruiz-Ramos M., Mendoza-Núñez V. M. (2014). 51 - Relationship between periodontal disease with oxidative stress markers in saliva in older adults.

[22] Baňasová L., Kamodyová N., Janšáková K. (2015). Salivary DNA and markers of oxidative stress in patients with chronic periodontitis.

[23] Baltacioglu E., Yuva P., Aydin G. (2014). Lipid peroxidation levels and total oxidant/antioxidant status in serum and saliva from patients with chronic and aggressive periodontitis. Oxidative stress index: a new biomarker for periodontal disease?.

[24] Trivedi S., Lal N., Mahdi A. A., Mittal M., Singh B., Pandey S. (2014). Evaluation of antioxidant enzymes activity and malondialdehyde levels in patients with chronic periodontitis and diabetes mellitus.

